# Supplementary material for: Drug repurposing for aging research using model organisms
Source: Aging Cell. 2017 Jun 16;16(5):1006–15. doi: 10.1111/acel.12626 (PMC5595691; doi:10.1111/acel.12626)
Supplement: Supplementary file 7 — Data S1 Zip‐Archive of all report cards. [file ACEL-16-1006-s007.zip › RC_754.pdf]

754

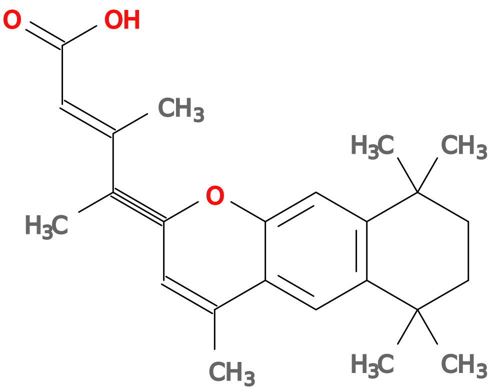

Database identifiers

ChEMBLCompound	CHEMBL109581

Ranking

	Rank	Score
Drosophila	531/697	0.223
C. elegans	NA	NA

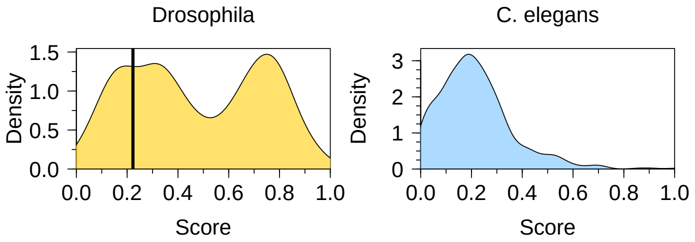

	Ageing implication	Domain conservation	Binding site conservation	Binding affinity	Bioavailability	Lipinski	Promiscuity	Purchasability	Drug approval	**Total**
Drosophila	0.624	0.649	0.811	0.925	(0.9)	-0.05	-0.0	0.0	0.0	0.223
C. elegans	NA	NA	NA	NA	NA	NA	NA	NA	NA	NA

Names

No synonyms found

Roles

ChEBI entry None has no roles

Status

Approved drug (according to ChEMBL)	No
Number of Rule of 5 violations	1
Binding affinity to original target in log units (RF-Score prediction)	7.51
Burns *C. elegans* bioavailability prediction	-4.01

Compound Target Characteristics

Retinoic acid receptor RXR-alpha

Best gene implication in ageing for this target family came from gene Q0VJ96 via mapping the annotation from RGD 3610 annotated in RGD 2014-03-11. Annotation GO 7568 (aging) was Inferred from Expression Pattern

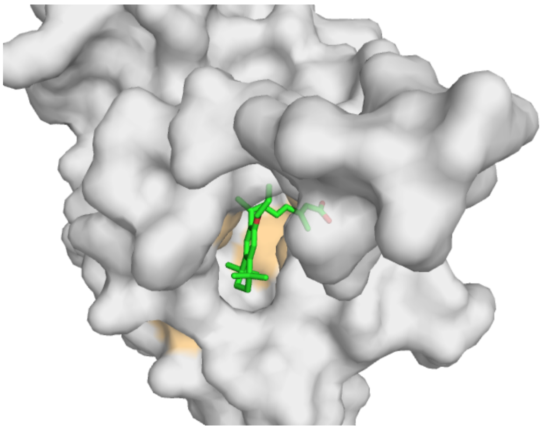

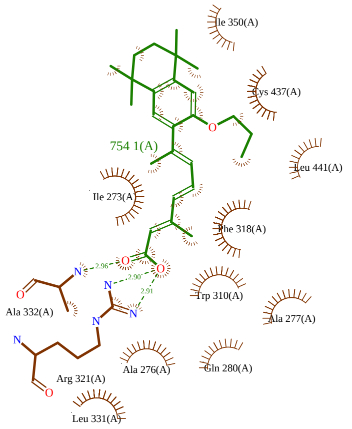

protein	amino acids contacts (binding site)
PDB:3a9e:chainA:P28700	I A A Q W F R L A I C L
tr:B3KY83:B3KY83_HUMAN	I A A Q W F R L A I C L
sp:P19793:RXRA_HUMAN	I A A Q W F R L A I C L
tr:F1D8Q5:F1D8Q5_HUMAN	I A A Q W F R L A I C L
tr:Q6P3U7:Q6P3U7_HUMAN	I A A Q W F R L A I C L
tr:Q0VJ96:Q0VJ96_RAT	I A A Q W F R L A I C L
tr:Q6LC96:Q6LC96_MOUSE	I A A Q W F R L A I C L
sp:P28700:RXRA_MOUSE	I A A Q W F R L A I C L
tr:A2AJP1:A2AJP1_MOUSE	I A A Q W F R L A I C L
sp:P20153:USP_DROME	L V V Q W F R L N I C L

	whole protein		domain-based		contact-based	
protein	ident	simil	ident	simil	ident	simil
PDB:3a9e:chainA:P28700	1.0	1.0	1.0	1.0	1.0	1.0
tr:B3KY83:B3KY83_HUMAN	0.78	0.78	1.0	1.0	1.0	1.0
sp:P19793:RXRA_HUMAN	0.97	0.99	1.0	1.0	1.0	1.0
tr:F1D8Q5:F1D8Q5_HUMAN	0.97	0.99	1.0	1.0	1.0	1.0
tr:Q6P3U7:Q6P3U7_HUMAN	0.87	0.88	1.0	1.0	1.0	1.0
tr:Q0VJ96:Q0VJ96_RAT	0.99	1.0	1.0	1.0	1.0	1.0
tr:Q6LC96:Q6LC96_MOUSE	0.94	0.94	1.0	1.0	1.0	1.0
sp:P28700:RXRA_MOUSE	1.0	1.0	1.0	1.0	1.0	1.0
tr:A2AJP1:A2AJP1_MOUSE	1.0	1.0	1.0	1.0	1.0	1.0
sp:P20153:USP_DROME	0.37	0.65	0.4	0.66	0.67	0.81

usp (FBgn0003964) associated phenotypes

circadian rhythm defective, germline clone, heat sensitive, hypoactive, lethal - all die before end of pupal stage, lethal - all die during P-stage, lethal - all die during first instar larval stage, maternal effect, neuroanatomy defective, partially, rescuable maternal effect, somatic clone, some die during pupal stage, touch sensitivity defective

(Information from FlyBase)

usp (UniProt:P20153) annotation

Function: Receptor for ecdysone. May be an important modulator of insect metamorphosis. Plays an important part in embryonic and post-embryonic development. Binds to ecdysone response elements (ECRES) such as in the promoter region of s15 chorion gene.

Subunit: Heterodimer of USP and ECR. Only the heterodimer is capable of high-affinity binding to ecdysone. (PubMed:8247157).
Subcellular location: Nucleus.
Domain: Composed of three domains: a modulating N-terminal domain, a DNA-binding domain and a C-terminal ligand-binding domain.

(Information from UniProt)
